# Pain-coping scale for children and their parents: a cross-sectional study in children with musculoskeletal pain

**DOI:** 10.1186/s12969-023-00791-1

**Published:** 2023-01-25

**Authors:** Maria Backström, Hanna Vuorimaa, Maarit Tarkiainen, Eliisa Löyttyniemi, Liisa Kröger, Kristiina Aalto, Katariina Rebane, Kati Markula-Patjas, Merja Malin, Sirja Sard, Paula Keskitalo, Katja Korkatti, Minna-Maija Grönlund, Milja Möttönen, Heini Pohjankoski, Maiju Hietanen, Johanna Kärki, Paula Vähäsalo

**Affiliations:** 1 Department of Paediatrics, Vaasa Central Hospital, Wellbeing services county of Ostrobothnia, Vaasa, Finland; 2 PEDEGO Research Unit, University of Oulu, Oulu, Finland; 3 The Finnish Center for Pediatric and Adolescent Pain Management and Research HUS, New Childrens Hospital, Helsinki, Finland; 4 New Childrens Hospital, Pediatric Research Center, Helsinki University Hospital and University of Helsinki, Helsinki, Finland; 5 Department of Biostatistics, University of Turku, Turku, Finland; 6 Department of Paediatrics, Kuopio University Hospital, Kuopio, Finland; 7 Department of Paediatrics, Tampere University Hospital, Tampere, Finland; 8 Centre for Child Health Research, Tampere University, Tampere, Finland; 9 Department of Paediatrics, Oulu University Hospital, Oulu, Finland; 10 Medical Research Center, Oulu University Hospital and University of Oulu, Oulu, Finland; 11 Department of Paediatrics, Central Ostrobothnia Central Hospital, Kokkola, Finland; 12 Department of Paediatrics, Turku University Hospital, Turku, Finland; 13 Department of Paediatrics, Päijät-Häme Central Hospital, Lahti, Finland; 14 Department of Children and Adolescents, Kanta-Häme Central Hospital, Hämeenlinna, Finland

**Keywords:** Child, Coping, Juvenile idiopathic arthritis, Musculoskeletal, Pain, Parent

## Abstract

**Background:**

In a chronic pain-causing disease such as juvenile idiopathic arthritis, the quality of coping with pain is crucial. Parents have a substantial influence on their children’s pain-coping strategies. This study aimed to develop scales for assessing parents’ strategies for coping with their children’s pain and a shorter improved scale for children usable in clinical practice.

**Methods:**

The number of items in the Finnish version of the pain-coping questionnaire for children was reduced from 39 to 20. A corresponding reduced scale was created for parental use. We recruited consecutive patients from nine hospitals evenly distributed throughout Finland, aged 8–16 years who visited a paediatric rheumatology outpatient clinic and reported musculoskeletal pain during the past week. The patients and parents rated the child’s pain on a visual analogue scale from 0 to 100 and completed pain-coping questionnaires and depression inventories. The selection process of pain questionnaire items was performed using factor analyses.

**Results:**

The average (standard deviation) age of the 130 patients was 13.0 (2.3) years; 91 (70%) were girls. Four factors were retained in the new, improved Pain-Coping Scales for children and parents. Both scales had 15 items with 2–5 items/factor. The goodness-of-fit statistics and Cronbach’s alpha reliability coefficients were satisfactory to good in both scaled. The criterion validity was acceptable as the demographic, disease related, and the depression and stress questionnaires correlated with the subscales.

**Conclusions:**

We created a shorter, feasible pain-coping scale for children and a novel scale for caregivers. In clinical work, the pain coping scales may serve as a visualisation of different types of coping strategies for paediatric patients with pain and their parents and facilitate the identification of families in need of psychological support.

**Supplementary Information:**

The online version contains supplementary material available at 10.1186/s12969-023-00791-1.

## Introduction

The support of the parents that are caring for a child with chronic pain is an important part of effective treatment. For instance, in juvenile idiopathic arthritis (JIA) that is a rheumatic disease with childhood onset, it has been shown that pain can remain a problem in a subgroup of patients despite clinical remission. This phenomenon occurs, at least in part, due to pain-specific beliefs regarding disability and harm as well as the pain-coping strategy of catastrophizing [1–3].

Coping refers to purposeful cognitive and behavioural actions that override the negative impact of stress [4]. The importance of coping with pain is well-recognised in children [5, 6]. Understanding the parental role in supporting a child in pain is increasing [7–9]; nevertheless, measurements of the precise mechanisms of parental pain-coping is less studied. Recently, Palit et al. proposed a multidomain pain resilience model to help to distinguish intraindividual and contextual factors that may enhance resilience and protective factors that mitigate adverse pain outcomes in children with pain [10]. Parental pain-coping may, at best, serve as a resilience factor and potentially protect the child from deterioration of functionality [10]. Parents have a crucial role in supporting the child’s adaptive or maladaptive pain-coping [11–13]. Thus, it appears essential to measure parental coping ability.

The pain-coping questionnaire (PCQ) for children and adolescents was first developed by Reid et al. [14]. The scale has been validated and modified in several countries [15–20]. Validation in Finnish was accomplished by Marttinen et al. [21]. Recently a short form of PCQ for children has been published [22] but ther are no existing questionnaire for assessing parents’ coping strategies when their child has pain, thus, there remained a need to create a corresponding scale for parents. Parents may enhance the child’s resilience with flexible coping abilities [10].

This study aimed to develop a valid, shortened PCQ for children because the use of long questionnaires may be exhausting for a child and inconvenient in clinical practice. A new PCQ was developed to assess parents’ coping strategies when their child has pain.

The aim of the study was, therefore, to assess the reliability and validity of the new questionnaires (named pain-coping scale for children [PCSped] and pain-coping scale for parents [PCSpar]) by testing the associations of the scales with demographic and disease factors together with comparison measures.

## Methodology

We reduced the number of items in the Finnish version of the PCQ from 39 to 20 [21] based on a discussion in a multidisciplinary team. Some of the questions were considered a bit difficult for children to understand and these where removed.

A corresponding scale with comparable items was created for parental use. The scales were named modified PCQ paediatric (mPCQped) and modified PCQ parental (mPCQpar) (Tables 1 and 2).

**Table 1:** The shortened pain coping questionnaire for children and adolescents (mPCQped). Response frequency of all patients (%) and mean and standard deviation (SD) of the scores in all patients and in children and adolescents separately

		Response frequency of all patiens (%)						All		Age 7.9–12.9			Age 13.0–18.3		
	WHEN I AM IN PAIN FOR A COUPLE OF HOURS OR DAYS I …	*N*	Never (1)	Rarely (2)	Sometimes (3)	Often (4)	Very often (5)	Mean	SD	*N*	Mean	SD	*N*	Mean	SD
Q1	ask adults about things related to my pain.	130	8.5	21.5	30.8	26.2	13.1	3.1	1.2	57	3.2	1.2	73	3.1	1.1
Q2	tell a friend how I feel.	130	11.5	30	23.1	26.9	8.5	2.9	1.2	57	2.8	1.2	73	3.0	0.9
Q3	start doing something.	129	7.7	17.7	29.2	33.8	10.8	3.2	1.1	56	3.3	1.2	73	3.2	1.1
Q4	worry that the pain will never stop.	130	33.8	23.8	27.7	7.7	6.9	2.3	1.2	57	2.3	1.3	73	2.3	1.1
Q5	talk with someone about how I feel.	129	10.8	23.8	25.4	27.1	12.3	3.1	1.2	56	3.1	1.3	73	3.0	0.9
Q6	do not pay attention to the pain.	130	6.2	28.5	33.8	25.4	6.2	3.0	1.0	57	3.1	1.1	73	2.8	1.2
Q7	quarrel, bicker or fight.	129	40	29.2	23.8	3.8	2.3	2.0	1.0	56	2.0	1.1	72	1.9	1.2
Q8	think all the time how much I am aching.	130	17.7	44.6	25.4	9.2	3.1	2.4	1.0	57	2.3	1.1	73	2.4	1.2
Q9	try to figure out different ways to relieve the pain.	130	13.1	16.2	24.6	33.1	13.1	3.2	1.2	57	3.0	1.3	73	3.3	1.1
Q10	explain to myself that there is nothing to worry about.	130	26.9	27.7	23.1	14.6	7.7	2.5	1.2	57	2.3	1.3	73	2.7	1.0
Q11	think that nothing will help.	130	38.5	36.9	12.3	10.0	2.3	2.0	1.1	57	1.8	1.0	73	2.2	1.1
Q12	get more information about how my body is working.	130	37.7	32.3	19.2	9.2	1.5	2.1	1.0	57	1.9	1.1	73	2.2	1.1
Q13	say to myself that soon everything will be all right.	130	33.1	20.8	25.4	16.2	4.6	2.4	1.2	57	2.2	1.2	73	2.6	1.1
Q14	start busying myself with something.	130	10.8	10	30	37.7	11.5	3.3	1.1	57	3.3	1.2	73	3.3	0.9
Q15	try not to think about the pain.	130	5.4	10	26.9	43.1	14.6	3.5	1.0	57	3.4	1.2	73	3.6	0.9
Q16	think that the pain will never ease off.	130	36.2	35.4	15.4	9.2	3.8	2.1	1.1	57	1.9	1.0	73	2.2	0.9
Q17	unburden my feelings to a friend.	130	26.2	29.2	23.8	15.4	5.4	2.4	1.2	57	2.2	1.2	73	2.6	1.0
Q18	explain to myself that I can overcome anything at all.	130	21.5	30	25.4	14.6	8.5	2.6	1.2	57	2.3	1.2	73	2.8	1.1
Q19	do something that will take the pain out of my mind.	130	11.5	10	26.9	37.7	13.8	3.3	1.2	57	3.3	1.3	73	3.3	1.1
Q20	worry about my pain almost all the time.	130	38.5	35.4	16.2	6.9	3.1	2.0	1.1	57	2.0	1.2	73	2.0	1.1

**Table 2:** The shortened pain coping questionnaire for parents (mPCQpar). Response frequency (%) of all parents and mean and standard deviation (SD) of the scores in all parents and in parents to children and adolescents separately

		Response frequency of all parents (%)						All		Age 7.9–12.9			Age 13.0–18.3		
	WHEN MY CHILD IS IN PAIN FOR A COUPLE OF HOURS OR DAYS I …	*N*	Never (1)	Rarely (2)	Sometimes (3)	Often (4)	Very often (5)	Mean	SD	*N*	Mean	SD	*N*	Mean	SD
Q1	ask the experts who treat my child about things related to my child’s pain.	127	1	14	32	40	12	3.5	0.9	56	3.6	0.9	71	3.4	0.9
Q2	tell a friend or spouse how I feel.	127	2	9	29	37	22	3.7	1.0	56	3.5	1.0	71	3.8	1.0
Q3	start doing something.	125	10	29	33	18	6	2.8	1.1	56	2.9	1.1	69	2.7	1.0
Q4	worry that my child’s pain will never stop.	127	11	32	25	24	6	2.8	1.1	56	2.8	1.1	71	2.8	1.2
Q5	talk with someone about how I feel.	127	2	19	27	40	11	3.4	1.0	56	3.2	0.9	71	3.6	1.0
Q6	try to focus on something else than my child’s pain.	126	14	24	29	28	3	2.8	1.1	55	3.0	1.1	71	2.7	1.1
Q7	quarrel or am tense and get nervous.	127	23	52	16	4	2	2.1	0.9	56	2.1	0.9	71	2.0	0.8
Q8	think all the time how much my child is aching.	127	12	43	23	16	3	2.5	1.0	56	2.6	1.0	71	2.5	1.0
Q9	try to figure out different ways to relieve my child’s pain.	127	1	15	1	45	36	4.2	0.7	56	4.2	0.7	71	4.2	0.7
Q10	say to myself that there is nothing to worry about my child.	127	12	19	38	22	8	2.9	1.1	56	3.2	1.2	71	2.8	1.0
Q11	think that nothing will help.	127	47	29	17	3	2	1.8	0.9	56	2.0	1.1	71	1.7	0.8
Q12	find out more information about the functioning of the body.	127	6	14	38	26	14	3.3	1.1	56	3.4	1.0	71	3.2	1.1
Q13	explain to myself that soon everything will be all right.	127	7	19	39	28	5	3.1	1.0	56	3.1	1.0	71	3.0	1.0
Q14	start pottering around with something with my child.	127	1	7	43	36	11	3.5	0.8	56	3.8	0.8	71	3.3	0.8
Q15	try not to think about my child’s pain.	126	9	27	37	23	2	2.8	1.0	56	3.0	1.0	70	2.7	0.9
Q16	think that my child’s pain will never ease off.	127	39	30	21	7	2	2.0	1.0	56	1.9	1.1	71	2.1	1.0
Q17	unburden my feelings to a friend or spouse.	127	6	13	27	35	17	3.4	1.1	56	3.3	1.1	71	3.5	1.1
Q18	assure myself that we can overcome anything at all.	127	2	9	20	42	25	3.8	1.0	56	4.0	0.9	71	3.6	1.0
Q19	do something that will take my child’s pain out of my mind.	127	8	26	42	18	5	2.9	1.0	56	3.0	1.0	71	2.8	0.9
Q20	worry about my child’s pain almost all the time.	127	25	40	19	11	3	2.2	1.1	56	2.2	1.0	71	2.3	1.1

Four certified translators independently translated and back-translated the mPCQped and mPCQpar into Swedish-Finnish and English-Finnish. The translators performing the back-translation did not take part in the first translation. An interdisciplinary team of translators, a psychologist (HV), a paediatric rheumatologist (PV) and two paediatricians (MB, MT) interpreted the results of the translations.

To validate the mPCQped and mPCQpar questionnaires, consecutive patients visiting the paediatric rheumatology outpatient clinic were recruited between October 2020 and July 2021. The study was conducted in five tertiary and four secondary hospitals evenly distributed throughout Finland. Children and adolescents aged 8 to 16 years with JIA or other musculoskeletal conditions causing pain were recruited if they had felt pain during the past week. The age 8 to 16 years was chosen whith the expectance that the children could read and understand the questionnaire and that the children would complete the questionnaire alone or together with the nurse. Families with insufficiency in the Finnish or Swedish languages were excluded. Data on age, gender, diagnosis, onset and duration of pain were collected. The patient and the accompanying parent rated the child’s (< 13 years) or adolescent’s (≥13 years) pain on a visual analogue scale (VAS) from 0 to 100. Patients completed the mPCQped, and parents completed the mPCQpar. Parents and adolescents were asked two questions dealing with stress, designed by one of the investigators (MT): how often during the last month they felt stress due to the disease or other reasons [Additional file 1] and a short two-item catastrophising questionnaire [23]. To measure depression, the children and adolescents completed the Finnish/Swedish version of the Children’s Depression Inventory (CDI) [24, 25], and the parents completed the Finnish/Swedish version of Beck’s Depression Inventory (BDI) [26]. The CDI score ranges from 0 to 52 and a score above 13 indicates depression. The BDI score ranges from 0 to 63 and scores 0–9 /10–18/19/29/30–63 indicate no/mild/moderate/severe depression respectively.

### Patients’ involvement statement

Ten patients and parents tested and commented on the mPCQ before use, otherwise the patients were not involved in the planning of the research.

### Statistics

Continuous variables were expressed as mean and standard deviation (SD) when normally distributed and with median and lower (Q1) and upper (Q3) quartiles when otherwise. The selection process of pain questionnaire items was performed using factor analyses. The estimation method was the maximum likelihood, and the rotation method was the oblimin method. Final communality above 0.3 for the items and rotated factor loading above 0.4 were criteria to move forward in the analyses. Criterion validity (i.e., the difference between factors in patients and parents according to gender and age of the patient [< or ≥ 13 years]) was tested using the t-test or Mann-Whitney U test as appropriate. The Hodges–Lehmann method was used to estimate median differences with 95% confidence intervals. The construct validity (i.e., the association of the factors of mPCQ and CDI, BDI, and pain VAS) was tested using Spearman’s correlation coefficient because some of the distributions were skewed. The construct validity was also assessed using the Kruskal-Wallis test to quantify differences in coping factors between three groups of patients and parents that experienced stress (1, never or rarely; 2, sometimes; 3, often or very often). *P*-values less than 0.05 (two-tailed) were considered statistically significant. Analyses were performed using SPSS Statistics, version 28.0.0.0. (190) (IBM, Armonk, NY, USA) and SAS System for Windows, version 9.4 (SAS Institute Inc., Cary, NC, USA).

## Results

Of the 153 families invited to the study, 130 (85%) attended. The average (SD) age was 13.0 (2.3) years; 70% were girls. Of the 130 contributing families, 119 were Finnish-speaking, and 11 were Swedish-speaking. The median (Q1–Q3) duration of pain was 14.0 (3.0–54.5) months. The median (Q1-Q3) patient and parent pain VAS were 37 (15–55) and 40 (20–59). The reasons for visiting the paediatric rheumatology outpatient clinic were JIA (*n* = 72), unspecific or postinfectious arthritis (*n* = 7), systemic connective tissue disease (*n* = 3), chronic non-bacterial osteomyelitis (*n* = 3), orthopaedic/orthognathic diagnosis (*n* = 4), or different musculoskeletal pain conditions (*n* = 41). The diagnoses of the 4 patients that had orthopedic conditions were M89.5 Osteolysis (*n* = 2) and M92.6 OCD (*n* = 2). The diagnoses of the 41 patients that had musculoskeletal pain were M25.5 arthralgia (*n* = 25), M30.3 polyarteritis nodosa (*n* = 1), M33.0 dermatomyositis (*n* = 1), M34.9 skleroderma (*n* = 1), M35.7 hypermobility syndrome (*n* = 1), M53.9 dorsalgia (*n* = 3), M79.6 limb pain (*n* = 5), M89.0 CRPS (*n* = 1) and M86.3 CNO (*n* = 3).

No clinically relevant differences were observed between the Finnish-speaking and Swedish-speaking families in the mPCQ (mPCQped and mPCQpar: data not shown). There were no significant differences in patient pain VAS or CDI scores between children and adolescents (Table 3). There were no significant differences in parents’ pain VAS and BDI between parents of children and adolescents. Of the 130 attending families 129–130 of the children (Table 1) and 125–127 of the parents (Table 2) had completed some or all of the questions in the mPCQ. Several steps in the exploratory factor analyses preceded the final factor analysis results for mPCQped and mPCQpar. These two scales were analysed separately.

**Table 3:** Pain rating, duration of pain and results of depression inventories. Patient and parent’s rating of the child’s pain on a visual analogue scale (VAS), the results of Children’s Depression Inventory (CDI) of the children, Beck’s depression Inventory (BDI) of the parents and duration of pain in months of all patients and of children (< 13.0 years) and adolescents (≥ 13.0 years) separately. Differences between children < 13.0 years and adolescents ≥13.0 years were tested by T-test* or Mann-Whitney U test^#^ as appropriate

	All			Age < 13.0			Age ≥ 13.0			95% CI
	N	Mean (SD)	Median (IQR)	N	Mean (SD)	Median (IQR)	N	Mean (SD)	Median (IQR)	
Patient pain VAS	128	37 (24)		55	36 (26)		73	37 (24)		−7.5 to 9.8*
Parent pain VAS (proxy)	121	38 (24)		55	40 (25)		66	37 (23)		−6.3 to 10.8*
CDI	127		5 (211)	55		4 (2–11)	72		6 (2–13)	−3.0 to1.0^#^
BDI	124		3 (1–6)	55		3 (0–6)	69		3 (1–6)	−1.0 to 1.0^#^
Duration of pain (months)	129		14 (3–54.5)	57		10.5 (2.2–28.5)	72		15 (3.1–60.0)	−11.1 to 1.3^#^

On the mPCQped and mPCQpar scale, all 20 items were first included in the analyses separately. The maximum likelihood estimation method with oblimin rotation method was used, which resulted in a four-factor solution. Five items from the children’s and parents’ questionnaires were removed from the final questionnaire ([children: Q1, Q6, Q7, Q9, Q12; Table 1]; (parents: Q1, Q7, Q9, Q12, Q14; Table 2]). The final factor analyses were executed with 15 items in both scales, and the four-factor solutions with oblimin rotation were retested. A satisfactory four-factor structure was accomplished using maximum likelihood analyses with oblimin rotation (Tables 4 and 5).

**Table 4:** Results from factor analyses in the children. Factor loadings and communalities based on a maximum likelihood estimation with oblimin rotation for 15 items and four factor solution from the modified pain coping questionnaire in children mPCQped (*n* = 130) and Eigenvalues, percentages of variance and cumulative percentages for the four factors

Item (used in the study)	Item number in final scale	Factor 1. Catastrohizing (CATped)	Factor 2. Positive cognitive distraction (PCDped)	Factor 3. Seeking social support (SSSped)	Factor 4. Behavioral distraction (BDped)	Communality
Q2. tell a friend how I feel.	1			0.79		0.63
Q3. start doing something.	2				0.61	0.39
Q4. worry that the pain will never stop.	3	0.82				0.66
Q5. talk with someone about how I feel.	4			0.52		0.40
Q8. think all the time how much I am aching.	5	0.58				0.38
Q10. explain to myself that there is nothing to worry about.	6		0.69			0.48
Q11. think that nothing will help.	7	0.72				0.54
Q13. say to myself that soon everything will be all right.	8		0.81			0.72
Q14. start busying myself with something.	9				0.91	0.83
Q15. try not to think about the pain.	10		0.46			0.35
Q16. think that the pain will never ease off.	11	0.74				0.57
Q17. talk about my feelings to a friend.	12			0.80		0.67
Q18. explain to myself that I can overcome anything at all.	13		0.76			0.64
Q19. do something that will take the pain out of my mind.	14		0.42			0.44
Q20. worry about my pain almost all the time.	15	0.73				0.52
Eigenvalue		4.31	2.91	1.45	1.26	
% of variance		28.72	19.38	9.67	8.43	
Cumulative %		28.72	48.10	57.77	66.20	
Cronbach alpha		0.85	0.82	0.77	0.72

**Table 5:** Results from factor analyses in the parents. Factor loadings and communalities based on a maximum likelihood estimation with oblimin rotation for 15 items and four factor solution from the modified pain coping questionnaire in parents (mPCQpar) (*n* = 130) and Eigenvalues, percentages of variance and cumulative percentages for the four factors

Item (used in the study)	Item number in final scale	Factor 1. Catastrophizing (CATpar)	Factor 2. Distraction (DISpar)	Factor 3. Seeking social support (SSS par)	Factor 4. Positive self statement (PSSpar)	Communality
Q2. tell a friend how I feel.	1			0.78		0.63
Q3. start doing something.	2		0.59			0.42
Q4. worry that my child’s pain will never stop.	3	0.79				0.64
Q5. talk with someone about how I feel.	4			0.81		0.70
Q6. try to focus on something else than my child’s pain.	5		0.93			0.82
Q8. think all the time how much my child is aching.	6	0.74				0.55
Q10. say to myself that there is nothing to worry about my child.	7				0.57	0.39
Q11. think that nothing will help.	8	0.61				0.39
Q13. explain to myself that soon everything will be all right.	9				0.92	0.84
Q15. try not to think about my child’s pain.	10		0.67			0.47
Q16. think that my child’s pain will never ease off.	11	0.79				0.65
Q17. unburden my feelings to a friend.	12			0.85		0.75
Q18. assure myself that we can overcome anything at all	13				0.54	0.37
Q19. do something that will take my child’s pain out of my mind.	14		0.66			0.51
Q20. worry about my child’s pain almost all the time.	15	0.77				0.59
Eigenvalue		3.66	2.94	2.22	1.43	
% of variance		24.38	19.63	14.80	9.55	
Cumulative %		24.38	44.00	58.81	68.36	
Cronbach alpha		0.86	0.81	0.86	0.69	

mPCQped scale: In the steps of analyses, the items reflecting catastrophising (CATped) loaded reliably on one separate factor (Table 4). The items in the factor seeking social support (SSS) were also stable, forming the SSSped factor in the final solution. Five items (‘say to myself that soon everything will be all right; I can overcome anything at all; there is nothing to worry about; try not to think about pain; do something that will take the pain out of my mind’) were unstable (i.e., loaded to different factors) during the analytical process as the items represented several aspects of pain-coping. In the final four-factor solution, the fit of the model was satisfactory. The factor was named positive cognitive distraction (PCDped). The fourth factor was called behavioural distraction (BDped), which included rational items to represent the content. The goodness-of-fit statistics were satisfactory (chi square = 754.5, *p* < 0.001, variance accounted = 66.2) and Cronbach’s alpha reliability coefficients were satisfactory (0.72–0.85) (Table 4).

mPCQpar scale: A four-factor solution was generated in the parental pain-coping scale (Table 5). In the analysis, catastrophising loaded reliably on one separate factor and was named CATpar (Table 5). The factor distraction (DISpar) included cognitive and behavioural items in the final factor solution. The other factors were ‘seeking social support’ (SSSpar) and ‘positive self-statement’ (PSSpar). The factor’s Cronbach’s alpha reliability coefficients were satisfactory (alpha = 0.70–0.86), and the goodness-of-fit statistics (variance accounted = 68.36) were good (Table 5). The new questionnaires were named pain-coping scale for children (PCSped) and parents (PCSpar) (Additional files 2–4).

Girls had significantly higher scores in CATped and SSSped than boys, and adolescents had significantly higher mean PCDped than children (Table 6). The parents of the adolescents had significantly lower mean in PSSpar and DISpar, than the parents of the children. There were no significant differences in the mean values of the other subscales between girls and boys or children and adolescents, between the parents of girls and boys, and between parents to children and adolescents.

**Table 6:** Criterion validity. Mean and SD of factors in children (ped) and parents (par) according to gender and age of the patient. Differences between girls and boys and between children < 13.0 years and adolescents ≥13.0 years were tested by T-test. The statistically significant results are marked in bold

	Al		Gender of patients					Age of patients				
			boys		girl		95% CI	< 13 years		≥13 years		95% CI
Factors	N	Mean (SD)	N	Mean (SD)	N	Mean (SD)		N	Mean (SD)	N	Mean (SD)	
Positive cognitive distraction (PCDped)	130	2.9 (0.9)	39	2.8 (1.0)	91	2.9 (0.8)	−0.47 to 0.39	57	2.7 (0.9)	73	3.0 (0.9)	−**0.63 to − 0.01**
Catastrophizing (CATped)	130	2.2 (0.9)	39	1.8 (0.8)	91	2.3 (0.8)	**−0.78 to − 0.15**	57	2.0 (0.9)	73	2.2 (0.8)	−0.50 to 0.10
Seeking social support (SSSped)	129	2.8 (1.0)	39	2.3 (0.9)	90	3.0 (1.0)	**−1.05 to − 0.34**	56	2.7 (1.1)	73	2.9 (0.9)	−0.50 to 0.21
Behavioral distraction (BDped)	129	3.3 (1.0)	38	3.1 (1.0)	91	3.3 (1.0)	−0.53 to 0.23	56	3.3 (1.0)	73	3.2 (1.0)	−0.28 to 0.42
Positive self statement (PSSpar)	127	3.3 (0.8)	38	3.1 (0.9)	89	3.3 (0.7)	−0.59 to 0.09	56	3.4 (0.8)	71	3.1 (0.8)	**0.04 to 0.60**
Catastrophizing (CATpar)	127	2.3 (0.8)	38	2.2 (0.8)	89	2.3 (0.8)	−0.38 to 0.25	56	2.3 (0.8)	71	2.3 (0.8)	−0.23 to 0.35
Seeking social support (SSSpar)	127	3.5 (0.9)	38	3.6 (0.9)	89	3.5 (0.9)	−0.30 to 0.49	56	3.4 (0.9)	71	3.6 (0.9)	−0.60 to 0.03
Distraction (DISpar)	124	3.0 (0.8)	37	2.9 (0.7)	87	3.0 (0.8)	−0.44 to 0.15	55	3.2 (0.8)	69	2.9 (0.7)	**0.05 to 0.58**

**Fig. 1:**
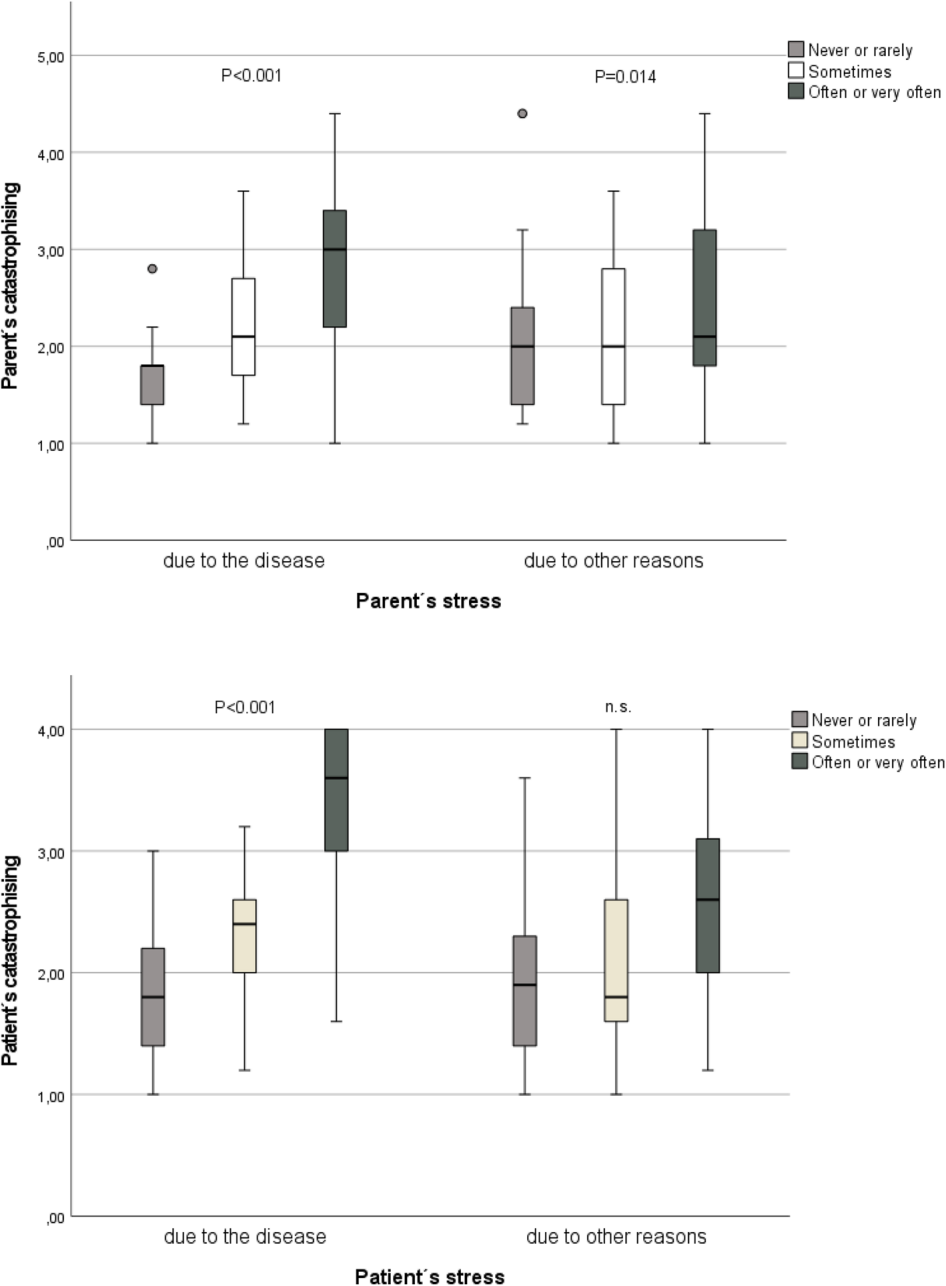
The catastrophizing in parents and patients according to stress. Tuley’s box plot together with mean values (x). The difference in coping factors between three groups of patients and parents that experienced stress (never or rarely, sometimes, often or very often) was tested with Kruskal-Wallis test

CATped correlated to some extent with CATpar and SSSped with SSSpar (Table 7). CATped and CATpar correlated strongly with the short two-item catastrophising questionnaire (Table 7, Table 8). CATped correlated to CDI and SSSped but not patient pain VAS or BDI. CATpar also correlated with BDI, parent pain VAS, and CDI. SSSpar was higher in parents experiencing stress due to the disease than in those who did not. CATped and CATpar were higher in parents and patients experiencing stress due to the disease and other reasons (Fig. 1).

**Table 7:** Construct validity of pain coping factors of the patients. Correlations (Spearman) between pain coping factors, depression inventory (CDI, BDI), pain score on a visual analogue scale (pain VAS), stress and a 2-item catastrophizing score in patients and parents

	PCDped			CATped			SSSped			BDped		
Children	N	R_s_	95% CI	N	R_s_	95% CI	N	R_s_	95%CI	N	R_s_	95% CI
Positive cognitive distraction (PCDped)	130	1.00		130	0.15	−0.02 to 0.32	129	0.40	0.24 to 0.54	129	0.36	0.20 to 0.51
Catastrophizing (CATped)	130	0.16	−0.02 to 0.32	130	1.00		129	0.26	0.09 to 0.42	129	0.07	−0.11 to 0.24
Seeking social support (SSSped)	129	0.40	0.24 to 0.54	129	0.26	0.09 to 0.42	129	1.00		128	0.24	0.06 to 0.40
Behavioral distraction (BDped)	129	0.36	0.20 to 0.51	129	0.07	−0.11 to 0.24	128	0.24	0.06 to 0.40	129	1.00	
CDI	127	−0.16	− 0.33 to 0.03)	127	0.49	0.34 to 0.61	126	−0.10	− 0.28 to 0.08	126	−0.19	− 0.35 to − 0.01
Patient pain VAS	128	0.01	−0.17 to 0.19)	128	0.08	−0.10 to 0.26	127	0.03	−0.15 to 0.21	127	−0.03	− 0.21 to 0.15
Patient’s stress due to the disease	66	0.05	−0.21 to 0.29	66	0.63	0.45 to 0.76	66	0.23	− 0.02 to 0.45	66	− 0.07	−0.32 to 0.18
Patient’s stress due to other factors	66	0.02	−0.23 to 0.27	66	0.44	0.21 to 0.62	66	0.09	−0.16 to 0.33	66	0.08	−0.17 to 0.32
2-item catastrophizing score ped	66	−0.03	−0.28 to 0.22	66	0.65	0.47 to 0.77	66	0.20	− 0.05 to 0.43	66	−0.12	−0.36 to 0.13
Parents												
Positive self statement (PSSpar)	127	0.05	−0.14 to 0.22	127	0.09	−0.08 to 0.27	126	0.05	−0.13 to 0.22	126	0.06	−0.12 to 0.24
Catastrophizing (CATpar)	127	−0.08	−0.26 to 0.10	127	0.19	0.01 to 0.36	126	0.15	−0.03 to 0.32	126	−0.15	−0.32 to 0.03
Seeking social support (SSSpar)	127	0.13	−0.05 to 0.31	127	0.03	−0.15 to 0.20	126	0.21	0.03 to 0.38	126	0.06	−0.12 to 0.24
Distraction (DISpar)	123	−0.06	−0.24 to 0.12	124	0.13	−0.30 to 0.05	123	0.00	−0.18 to 0.18	123	0.10	−0.08 to 0.27
BDI	124	−0.13	−0.30 to 0.06	124	0.03	−0.15 to 0.21	123	0.02	−0.17 to 0.20	123	−0.08	−0.26 to 0.11
Parent pain VAS	121	0.16	−0.03 to 0.33	121	0.18	0.00 to 0.35	120	0.16	−0.03 to 0.34	120	0.02	−0.16 to 0.21
Parent’s stress due to the disease	126	−0.09	− 0.26 to 0.09	126	0.08	−0.10 to 0.25	125	0.04	−0.14 to 0.22	125	−0.13	−0.30 to 0.06
Parent’s stress due to other factors	126	−0.09	− 0.27 to 0.09	126	0.03	−0.15 to 0.21	125	−0.01	− 0.19 to 0.17	125	0.02	−0.16 to 0.20
2-item catastrophizing score par	123	−0.12	− 0.30 to 0.06	123	0.12	−0.06 to 0.30	122	0.01	−0.17 to 0.19	122	−0.10	−0.27 to 0.09

**Table 8:** Construct validity of pain coping factors of the parents. Correlations (Spearman) between pain coping factors of the parents, depression inventory (CDI, BDI), pain score on a visual analogue scale (pain VAS), stress and a 2-item catastrophizing score in patients and parents

	PSSpar			CATpar			SSSpar			DISpar		
Children	N	R_s_	95% CI	N	R_s_	95% CI	N	R_s_	95% CI	N	R_s_	95% CI
CDI	124	−0.02	−0.20 to 0.16	124	0.21	0.03 to 0.38	124	−0.09	−0.27 to 0.10	120	−0.04	− 0.23 to 0.14
Patient pain VAS	125	0.00	−0.19 to 0.18	125	0.16	−0.02 to 0.33	125	0.04	−0.14 to 0.22	121	0.07	−0.12 to 0.25
Patient’s stress due to the disease	64	0.05	−0.20 to 0.30	64	0.23	−0.03 to 0.46	64	−0.07	− 0.32 to 0.19	61	−0.01	− 0.28 to 0.24
Patient’s stress due to other factors	64	0.20	−0.06 to 0.43	64	0.07	−0.18 to 0.32	64	−0.16	− 0.40 to 0.10	61	0.01	−0.26 to 0.26
2-item catastrophizing score ped	64	0.07	−0.19 to 0.32	64	0.17	−0.09 to 0.41	64	−0.15	−0.39 to 0.11	61	−0.09	− 0.34 to 0.18
Parents												
Positive self statement (PSSpar)	127	1.00		127	0.01	−0.18 to 0.18	127	0.17	−0.01 to 0.34	123	0.36	0.19 to 0.51
Catastrophizing (CATpar)	127	0.00	−0.18 to 0.18	127	1.00		127	0.09	−0.09 to 0.27	123	0.06	−0.12 to 0.24
Seeking social support (SSSpar)	127	0.17	−0.01 to 0.34	127	0.09	−0.09 to 0.27	127	1.00		123	0.25	0.07 to 0.41
Distraction (DISpar)	123	0.36	0.19 to 0.51	123	0.06	−0.12 to 0.24	123	0.25	0.07 to 0.41	123	1.00	
BDI	123	−0.05	−0.23 to 0.13	123	0.33	0.16 to 0.48	123	0.02	−0.17 to 0.20	120	0.03	−0.15 to 0.22
Parent pain VAS	120	−0.15	−0.32 to 0.04	120	0.23	0.05 to 0.37	120	0.04	−0.15 to 0.22	116	0.00	−0.18 to 0.19
Parent’s stress due to the disease	126	−0.05	−0.23 to 0.13	126	0.53	0.39 to 0.65	126	0.25	0.08 to 0.42	122	0.12	−0.06 to 0.30
Parent’s stress due to other factors	126	−0.02	−0.20 to 0.62	126	0.27	0.09 to 0.43	126	0.00	−0.19 to 0.18	122	0.02	−0.17 to 0.20
2-item catastrophizing score par	123	−0.13	−0.30 to 0.06	123	0.57	0.43 to 0.68	123	0.09	−0.09 to 0.27	120	0.08	−0.10 to 0.27

There was a strong correlation between patient and parent pain VAS (r_s_ = 0.70, 95% CI 0.59 to 0.79). The parent pain VAS correlated with BDI (r_s_ = 0.30, 95% CI 0.12 to 0.46); however, the patient pain VAS did not correlate with CDI or BDI. There was a strong correlation between BDI and parental stress due to disease (r_s_ = 0.46, 95% CI 0.30 to 0.59) and parental stress due to other factors (r_s_ = 0.50, 95% CI 0.36 to 0.64). The correlations between CDI and stress due to the disease and other factors were also strong in adolescents (r_s_ = 0.65, 95% CI 0.47 to 0.72, r_s_ = 0.56, 95%, CI 0.36 to 0.71).

## Discussion

The present study successfully created instruments for clinical use to measure pain-coping with an abbreviated scale for children and a novel scale for parents.

The study demonstrated adequate internal consistency measured by an alpha coefficient and reliability for PCSped and PCSpar. Four subscales were detected in PCSped (CATped, SSSped, BDped, PCDped) and PCSpar (CATpar, SSSpar, PSSpar, and DISpar). This study also demonstrated that the questionnaires have good psychometric properties (e.g., reliability). The criterion validity was acceptable as the demographic, disease related, and the comparison questionnaires correlated with PCSpar and PCSped subscales.

In agreement with earlier studies [21, 27], CATped and CATpar were strongly associated with elevated levels of depressive symptoms (measured by CDI and BDI) and with the level of stress. The content of the catastrophising factor included items reflecting aspects such as rumination, helplessness, and heightened threat. In our study, CATpar was associated with depressive symptoms in children and parents. Similarly, Caes et al. found that parents with catastrophising thinking prioritise pain control over active engagement. In acute pain situations, parental catastrophising might be functional and foster pain relief; however, in the long run, perseverance in pain control may become dysfunctional [28]. It was recently found that parental catastrophising has a substantial impact on the functional disability of the child and that parental protective behaviour independently slows child’s functional improvement [29].

In the current study of the parental factors, catastrophising was a robust factor. The entire picture of parental coping is likely to be complicated. A child’s pain may provoke a parental need to help the child. Thus, coping can be seen as a dyadic process in which several reciprocal aspects interact regarding a child in pain. Our sample’s median duration of pain was one year, which most likely affected the whole family. However, the parents and the patients were not severely depressed. This phenomenon may have limited the use of depressive symptoms as a measure of criterion validity because the variability of the scale was low.

Parents require different aspects of coping to support a child [7–10]. On the other hand, children tend to have fears, catastrophising thoughts, magnification of possible awful consequences of pain, and less experience of coping attempts [30]. It follows that the content of child’s and adolescent’s coping styles with pain differs from that of adults. This was also a finding of the current study, as the item content of the subfactors in the parental and child scales differed slightly.

There was a correlation between CATped and CATpar and SSSped and SSSpar. Parents who are the most important adults in the life of a developing child, might shape their child’s functioning with pain in several ways. Parental coping (e.g., optimal psychological flexibility or parental distress) may serve as a resilience or risk mechanism for a child [31]. Recently, Stone and Wilson [32] introduced a model for transmitting chronic pain from parents to offspring. One aspect of the model is pain-specific social learning, through which children may learn pain-coping by modelling their parents. In the current cross-sectional study, parental pain symptoms were not studied, such that evaluation of the transmission model was not possible. However, pain catastrophising, a non-adaptive coping style, may be socially passed for the child e.g., by restricting potentially painful activities of the child or by communicating high threat information about pain in the family [32]. Some support has been found for the social learning perspective in families with pain symptoms [33]. In turn, the daily fluctuation of parenting stress appears to influence their pain-coping [7].

In JIA, disease activity is measured by Juvenile Arthritis Disease Activity Score (JADAS) [34]. The child’s overall well-being assessed by the parent/patient (PaGA) is one of four parameters in JADAS. PaGA has been shown to correlate strongly with a parent’s assessment of their child’s pain [35]. Because in this study, the parents´ assessments of their child’s pain correlated with the CATpar, we believe that the catastrophising of the patient and parent should be considered when evaluating the overall situation at rheumatologic visits. Pain is a multidimensional and stressful experience including sensory, affective and cognitive components. Cognitive and behavioral processing with pain experience may be rigid and insufficient, enabling minor alleviation in stress and pain [22]. Pain coping scales, such as PCSped and PCSpar may function as qualitative clinical instruments as well as providing data for visualization of different coping resources the child and the parent has in clinical settings. By understanding the pain coping profile and individuals needs of the patient and the parent, it is possible to optimize the treatment modalities in timing and length. Specific understanding of the content of coping of an individual may speed up choosing appropriate treatment for them. Because CATped and CATpar subscales showed a significant correlation with the two-item catastrophising scale in children, the shorter two-item catastrophising scale could be used to evaluate the catastrophising situation in both patients and parents swiftly. However, we believe that using the PCS might be beneficial as it provides information on coping strategies other than catastrophising.

There are some limitations to our study. The study was cross-sectional, and this structure does not enable conclusions to be drawn about the direction or causality of the relationships between pain-coping and clinical data. In the near future the scales need to be further validated through a prospective study, as pain coping strategies for patients and parents may be influenced by the duration of the disease, duration of the high/moderate disease activity, the treatment success, even by the time needed to reach remission. In data collection, coping-related factors such as optimism and psychological flexibility would have strengthened the current results because some subfactors of the scales reflected the aspect of active coping. Adding a generic quality-of-life coping-related questionnaire would have clarified the meaning of pain-coping in children’s and adolescents’ lives. The duration of pain was under 1 month only in 24 patients and the results of our finding might have been different in a population only with acute pain. The stress questionnaire has not been tested, published or routinely used which also can be a limitation of our study. The study would have benefitted from a larger, international sample size. There were only 10 answers in Swedish, a minority language in Finland, and conclusions are difficult to draw about this patient group.

The strength of our study was that pain-coping was investigated in children and parents, and thus we found that the coping strategies differed somewhat in parents and children. Moreover, children and adolescents with musculoskeletal pain were systematically recruited from secondary and tertiary centres throughout Finland, the patients where consecutive patients and the spectrum of their diagnoses was typical for rheumatologic outpatient clinics in Finland. The sample-size was adequate based on current recommendations [36].

In the future, it would be helpful to validate the questionnaires in an international population to determine whether cultural differences would impact the results. Further validation of the questionnaire in a prospective setting would help to explain the causal relationship or its direction between pain-coping and clinical data. Shortly, the association of subfactors reflecting active engagement (e.g., positive cognitive distraction and seeking social support) could be tested with optimistic processes or psychological flexibility.

## Conclusions

The current study demonstrated the feasibility of the PCSpar and PCSped scales. The questionnaires can be used as qualitative clinical instruments to identify pain-coping strategies of children and adolescents and their parents. In clinical work, PCSped and PCSpar may serve as a visualisation of different types of coping resources for paediatric patients with pain and their parents and facilitate the identification of families in need of psychological support.

## Supplementary Information

**Additional file 1.** Stress questionnaire. Stress questionnaire for adolescents and parents.

**Additional file 2.** Pain coping scale for a child. Pain Coping Scale (PCSped).

**Additional file 3.** Pain coping scale for a parent. Pain Coping Scale (PCSpar).

**Additional file 4.** User manual for pain coping scale for children (PCSPed) and parents (PCSPar).

## Data Availability

Deidentified individual participant data (including data dictionaries) will be made available, in addition to study protocols, the statistical analysis plan, and the informed consent form. The data will be made available upon publication until July 2031 to researchers who provide a methodologically sound proposal for use in achieving the goals of the approved proposal. Proposals should be submitted to maria.backstrom@ovph.fi.

## Abbreviations

BD: behavioural distraction
BDI: Beck’s Depression Inventory
CAT: catastrophising
CDI: Children’s Depression Inventory
DIS: distraction
mPCQped: modified PCQ paediatric
JIA: juvenile idiopathic arthritis
mPCQ: modified pain coping questionnaire
par: for parents
PCD: positive cognitive distraction PCQ, pain-coping questionnaire
PCS: pain-coping scale
ped: for children
PSS: positive self-statement
SSS: seeking social support
VAS: visual analogue scale
