# Supplementary material for: Pain-coping scale for children and their parents: a cross-sectional study in children with musculoskeletal pain
Source: Pediatr Rheumatol Online J. 2023 Jan 25;21:9. doi: 10.1186/s12969-023-00791-1 (PMC9875488; doi:10.1186/s12969-023-00791-1)
Supplement: Supplementary file 1 — Additional file 1. Stress questionnaire. Stress questionnaire for adolescents and parents. [file 12969_2023_791_MOESM1_ESM.pdf]

Additional file 1. Stress questionnaire.

Stress refers to a situation where a person is faced with so many challenges or demands on him/herself that his/her available resources are stretched out or exceeded. A stressed-out person may be nervous or irritated about not being able to control things or his/her own life.

	Never	Rarely	Sometimes	Often	Very often
In the past month, how often have you felt this way due to the disease?	1	2	3	4	5
In the past month, how often have you felt this way due to other reasons?	1	2	3	4	5
