# Supplementary material for: Pain-coping scale for children and their parents: a cross-sectional study in children with musculoskeletal pain
Source: Pediatr Rheumatol Online J. 2023 Jan 25;21:9. doi: 10.1186/s12969-023-00791-1 (PMC9875488; doi:10.1186/s12969-023-00791-1)
Supplement: Supplementary file 2 — Additional file 2. Pain coping scale for a child. Pain Coping Scale (PCSped). [file 12969_2023_791_MOESM2_ESM.pdf]

FOR A CHILD

Pain coping Scale (PCSped)

©Backström, Vuorimaa, Vähäsalo et al. 2022

Name:____

Identity code:____

Everyone has sometimes felt pain for a couple of hours or even longer.
The following list includes things that people may say, do or think when they are in pain.
We wish to know what you do when you are in pain for a couple of hours or days.
In each question, circle the number according to how often you act or think in the way in question:
1=never, 2=rarely, 3=sometimes, 4=often or 5=very often
We may ask some things several times intentionally. Do not let it disturb you.

	WHEN I AM IN PAIN FOR A COUPLE OF HOURS OR DAYS I					
		Never	Rarely	Sometimes	Often	Very often
1	tell a friend how I feel.	1	2	3	4	5
2	start doing something.	1	2	3	4	5
3	worry that the pain will never stop.	1	2	3	4	5
4	talk with someone about how I feel.	1	2	3	4	5
5	think all the time how much I am aching.	1	2	3	4	5
6	explain to myself that there is nothing to worry about.	1	2	3	4	5
7	think that nothing will help.	1	2	3	4	5
8	say to myself that soon everything will be all right.	1	2	3	4	5
9	start busying myself with something.	1	2	3	4	5
10	try not to think about the pain.	1	2	3	4	5
11	think that the pain will never ease off.	1	2	3	4	5
12	talk about my feelings to a friend.	1	2	3	4	5
13	explain to myself that I can overcome anything at all.	1	2	3	4	5
14	do something that will take the pain out of my mind.	1	2	3	4	5
15	worry about my pain almost all the time.	1	2	3	4	5
