# Supplementary material for: Pain-coping scale for children and their parents: a cross-sectional study in children with musculoskeletal pain
Source: Pediatr Rheumatol Online J. 2023 Jan 25;21:9. doi: 10.1186/s12969-023-00791-1 (PMC9875488; doi:10.1186/s12969-023-00791-1)
Supplement: Supplementary file 3 — Additional file 3. Pain coping scale for a parent. Pain Coping Scale (PCSpar). [file 12969_2023_791_MOESM3_ESM.pdf]

FOR A CAREGIVER

Pain coping scale (PCSpar)

©Backström, Vuorimaa, Vähäsalo et al. 2022

Child's name:____

Child's identity code:____

Respondent (circle):

Mother Father Somebody else, who?

Every child has sometimes felt pain for a couple of hours or even longer.
The following list includes things that parents may say, do or think when their child is in pain.
We are interested in how you act or think when your child is in pain for a couple of hours or days.
In each question, circle the number according to how often you act or think in the way in question:
1=never, 2=rarely, 3=sometimes, 4=often or 5=very often.
We may ask some things several times intentionally.
Do not let it disturb you.

	WHEN MY CHILD IS IN PAIN FOR A COUPLE OF HOURS OR DAYS I …	Never	Rarely	Sometimes	Often	Very often
1	tell a friend or spouse how I feel.	1	2	3	4	5
2	start doing something.	1	2	3	4	5
3	worry that my child's pain will never stop.	1	2	3	4	5
4	talk with someone about how I feel.	1	2	3	4	5
5	try to focus on something else than my child's pain.	1	2	3	4	5
6	think all the time how much my child is aching.	1	2	3	4	5
7	say to myself that there is nothing to worry about my child.	1	2	3	4	5
8	think that nothing will help.	1	2	3	4	5
9	explain to myself that soon everything will be all right.	1	2	3	4	5
10	try not to think about my child's pain.	1	2	3	4	5
11	think that my child's pain will never ease off.	1	2	3	4	5
12	unburden my feelings to a friend or spouse.	1	2	3	4	5
13	assure myself that we can overcome anything at all.	1	2	3	4	5
14	do something that will take my child's pain out of my mind.	1	2	3	4	5
15	worry about my child's pain almost all the time.	1	2	3	4	5
