# Supplementary material for: Pain-coping scale for children and their parents: a cross-sectional study in children with musculoskeletal pain
Source: Pediatr Rheumatol Online J. 2023 Jan 25;21:9. doi: 10.1186/s12969-023-00791-1 (PMC9875488; doi:10.1186/s12969-023-00791-1)
Supplement: Supplementary file 4 — Additional file 4. User manual for pain coping scale for children (PCSPed) and parents (PCSPar). [file 12969_2023_791_MOESM4_ESM.pdf]

User manual for pain coping scale for children (PCSPed) and parents (PCSPar)

Copyright: If PCSped or PCSpar is used in research leading to a publication, this original publication should be quoted (Backström et al. Pain coping scale for children (PCSped) and their parents (PCSpar) - A cross-sectional study in pediatric patients with juvenile idiopathic arthritis or other cause of musculoskeletal pain. (Pediatric rheumatology DOI: 10.1186/s12969-023-00791-1). Otherwise the use of the scales is free.

The scale is not a diagnostic tool. PCSped and PCSpar are designed to help clinicians to get a more comprehensive picture of the families´ coping strategies when a child/adolescent has pain.

The child should fill in the questionnaire **alone** (without parents) if possible and otherwise assisted by a nurse or a doctor when needed.

Scaling

PCSped is composed of 4 factors including following items

Factors	Items
Catastrophising (CATped)	3.worry that the pain will never stop.
	5.think all the time how much I am aching.
	7.think that nothing will help.
	11.think that the pain will never ease off.
	15.worry about my pain almost all the time.
Positive cognitive distraction (PCDped)	6.explain to myself that there is nothing to worry about.
	8.say to myself that soon everything will be all right.
	10.try not to think about the pain.
	13.explain to myself that I can overcome anything at all.
	14.do something that will take the pain out of my mind.
Seeking social support (SSSped)	1.tell a friend how I feel.
	4.talk with someone about how I feel.
	12.talk about my feelings to a friend.
Behavioral distraction (BDped)	2.start doing something.
	9.start busying myself with something.

PCSpar is composed of 4 factors including following items

Factors	Items
Catastrophisng (CATpar)	3.worry that my child's pain will never stop.
	6.think all the time how much my child is aching.
	8.think that nothing will help.
	11.think that my child's pain will never ease off.
	15.worry about my child's pain almost all the time.
Distraction (DISpar)	2.start doing something.
	5.try to focus on something else than my child’s pain.
	10.try not to think about my child's pain.
	14.do something that will take my child's pain out of my mind.
Seeking social support (SSSpar)	1.tell a friend how I feel.
	4.talk with someone about how I feel.
	12.unburden my feelings to a friend.
Positive self statement (PSSpar)	7.say to myself that there is nothing to worry about my child.
	9.explain to myself that soon everything will be all right.
	13.assure myself that we can.

Scoring of domains

- Mean values of items are calculated for each domain. For mean values in the study population see Table 6.
- Two (out of five) missing values are allowed in CATped, PCDped and CATpar, one (out of four or three) in SSSped, DISpar, SSSpar and PSSpar but no missing values allowed in BDped.
- Every item is weighted equally.
- Cut-off levels have not been determined.
